# Genomic Evidence of Low Contemporary Effective Population Size and Southern Genetic Reservoirs in an Island Endemic Epiphytic Orchid of Taiwan

**DOI:** 10.1002/ece3.72920

**Published:** 2026-01-14

**Authors:** Wei‐Yun Chen, Yi Sun, Shu‐Ping Wu, Jen‐Pan Huang

**Affiliations:** ^1^ Biodiversity Research Center Academia Sinica Taipei Taiwan; ^2^ Institute of Ecology and Evolutionary Biology National Taiwan University Taipei Taiwan; ^3^ Department of Earth and Life Science University of Taipei Taipei Taiwan

**Keywords:** conservation genetics, ddRADseq, effective population size, *Holcoglossum pumilum*, overcollection, reintroduction

## Abstract

Orchids are traded globally, where wild populations can be threatened by overharvesting, habitat loss, and climate change. Many orchid species lack *ex situ* protection, such as botanical gardens, making in situ genetic studies of wild populations critical to inform conservation plans. Taiwan is a center for orchid diversity and has witnessed declines in wild orchids. *Holcoglossum pumilum*, an island endemic epiphytic species, is popular among citizen scientists and shows potential for public engagement in biodiversity conservation. This study aims to evaluate the genetic diversity, population structure, and adaptive potential of wild *Holcoglossum pumilum* in Taiwan, not only to serve as an example for public outreach but also to inform conservation policy. We collected 64 plants from 18 sites across Taiwan and used ddRADseq to generate genome‐wide SNP data. We found higher genetic diversity in southern populations than in northern ones. While evidence for genetic clustering and isolation by distance was limited, we detected significant genotype–environment associations, especially with annual precipitation. Demographic reconstructions suggested a pre‐LGM expansion followed by a plateau. Contemporary Ne estimates varied by data filtering strategies but were mostly below 500. Our study demonstrates the critical role of genomic data not only in revealing the evolutionary origin of genetic diversity but also in guiding conservation plans. Specifically, we argued that the seasonal monsoon and the mountainous landscape of Taiwan may have shaped a latitudinal gradient of genetic diversity in *H. pumilum* . As a result, southern populations exhibit greater genetic diversity, which makes them priorities for conservation management.

## Graphical abstract

Genome‐wide data from ddRADseq revealed a latitudinal gradient in genetic diversity in a Taiwanese endemic orchid. This study shows that genomic data not only can help us understand the evolutionary origins of genetic diversity in a species but also uncover its genetic reservoir for future conservation planning.

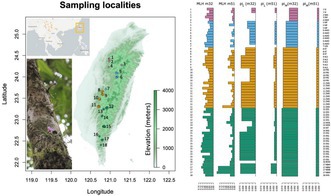

## 1 Introduction

Orchids are among the most internationally traded plants, generating hundreds of millions of dollars in revenue annually (Hinsley et al. 2018). The global orchid trade involves both commercially cultivated species and wild‐sourced orchids, the latter of which may or may not have been harvested legally. Orchid collectors and breeders may harvest wild orchids as collateral damage due to the demands of the large orchid trade market. In particular, rare orchids are often of special interest to private collectors and nurseries, often in the absence of established regulations or even basic biological information necessary for their sustainable harvest (Subedi et al. 2013). Recent studies have further documented population declines in wild orchid species directly linked to increased collection pressure driven by market demand (Fay 2018; Ticktin et al. 2023; Pitogo and Saavedra 2024). In response, both local and international conservation efforts have gained urgency, not only due to overharvesting, but also because of the increasing threats posed by habitat loss and climate change (Swarts and Dixon 2009; Wraith et al. 2020). Unlike commercially important cultivated ornamental plants, including orchids, that their genetic diversity may be conserved *ex situ* through global botanical garden and nursery networks (Swarts and Dixon 2009; Murrell et al. 2025), many wild orchid species lack such genetic reservoirs to support their future evolvability. Therefore, studying the genetic diversity of wild populations in situ is essential to assess whether local adaptation or genetic erosion has occurred in the recent past. This genetic information is critical for informing future conservation programs (DeSalle and Amato 2004; Hoban et al. 2013; Tkach and Watson 2023), and especially for wild orchids where many programs are promoting and monitoring the results of reintroduction (Reiter et al. 2016; Zhang et al. 2025).

The island of Taiwan has not only been a major center exporting cultivated orchids (Fay 2018; Hinsley et al. 2018), but also the genetic and geographical origin of the progenitors of many commercially important orchids, owing to its high diversity (Lin and Liu 2025). However, due to the high commercial value of ornamental orchids, wild populations of many species have rapidly declined and may have become nearly extinct in recent decades. Because of their social and economic importance, recent efforts, which include the identification of genetic reservoirs and reintroduction programs, have been implemented or planned for flagship species such as *Phalaenopsis amabilis*, *P. equestris* , and *Vanda lamellata*. Specifically, recent efforts to protect and restore forest habitats in Taiwan (Chen et al. 2019) may facilitate the reintroduction of orchids into their original, but locally extinct, ranges. Nevertheless, a large number of wild orchid species also experienced intense historical and even ongoing harvesting, and the current status of their population genetic diversity remains largely understudied (Chao et al. 2021). The genetic diversity of wild orchids in Taiwan, on the other hand, may provide critical information into how different populations respond to different levels and histories of anthropogenic disturbance (such as in De Vivo et al. 2023; Shen, Le, et al. 2025), and whether genetic diversity, and in particular adaptive genetic diversity, still exists within these populations to inform future policymaking.

**FIGURE 1:**
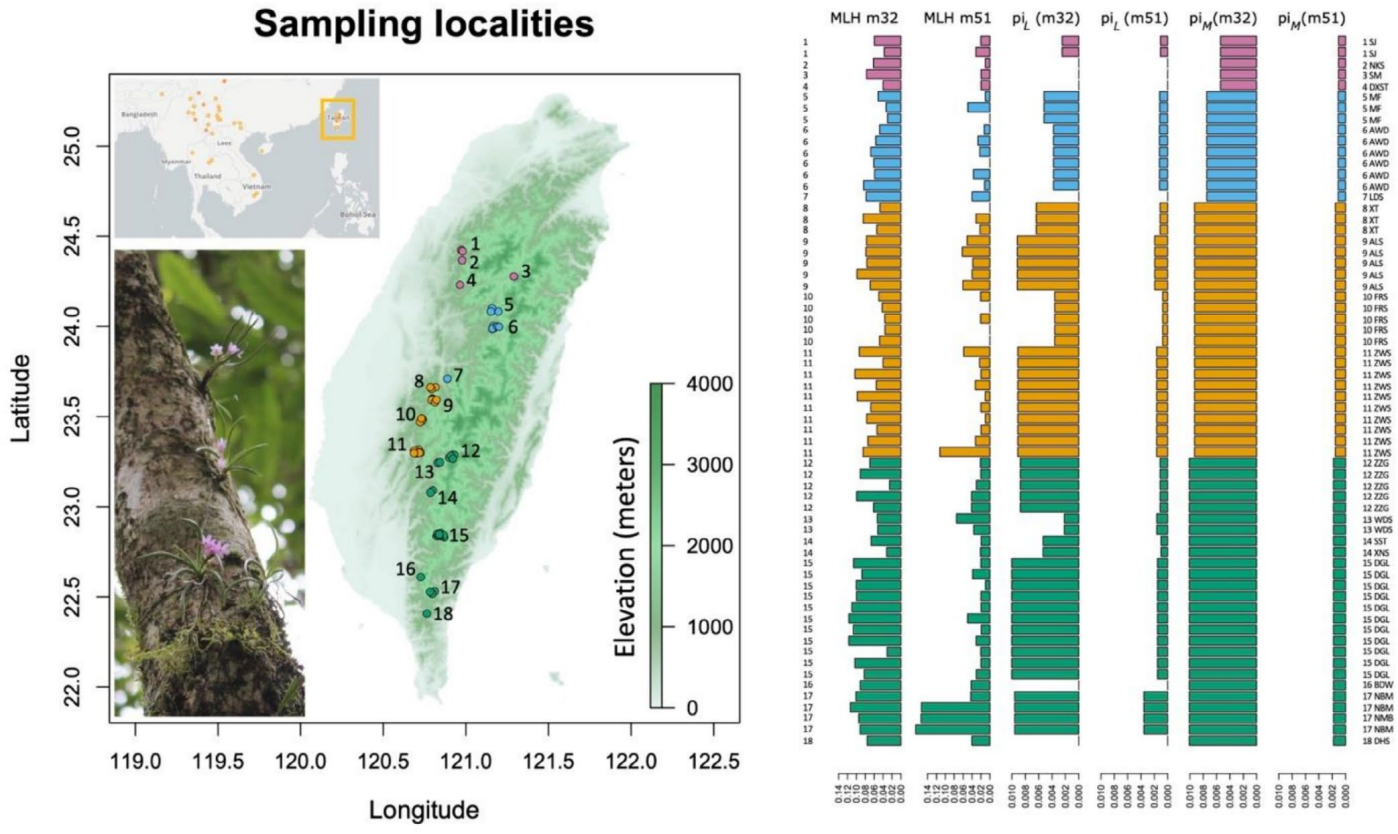
A map of Taiwan displays the sampling localities and population genetic summary statistics for each individual and population. An inset map in the top left corner shows Taiwan's geographical location in East and Southeast Asia with colored dots indicating collection records of *Holcoglossum* orchids (data retrieved on May/01, 2025 from GBIF). Samples and localities from four distinct mountain ranges are color‐coded as follows: Purple for the Xueshan Mountain Range (XSM), blue for the Central Mountain Range (CMR), gold for the Alishan Mountain Range (ALS), and green for the Southern Central Mountain Range (SMR). The genetic summary statistics include MLH, multilocus heterozygosity; pi_*L*_, estimated nucleotide diversity (π) for each local population; note that for populations with only one representative sample, pi_*L*_ values were not estimated; pi_*M*_, Estimated nucleotide diversity (π) for samples from each mountain range. m32 and m51 denote the datasets used for estimating the summary statistics. Numbers (1–18) next to the sampling localities correspond to those in the population summary statistics next to each individual. Details of the sampling localities, including GPS coordinates, population names, and collection dates, are available in Data S3. The map also includes an image showing *H. pumilum* orchids in their natural habitats (photos taken by J.‐P. Huang in the Xuejian Forest Recreation Area [SJ, population number 1 in the map] on February 10, 2025).

*Holcoglossum pumilum* (Hayata) is a miniature epiphytic orchid, endemic to the island of Taiwan, and distributed in mid‐ to high‐elevation forest habitats (Figure 1; Xiang et al. 2012; Lin and Liu 2025). The species is currently listed as Least Concern in the Taiwanese flora; however, we want to note that conservation status, or perceived extinction risk based on Red List criteria, often does not correlate with the actual genetic diversity nor the future evolutionary potential of a species (Schmidt et al. 2023). Furthermore, although it is not a commercially harvested or widely cultivated species, it is still regularly sold in local markets or online at prices about 10 US dollars per individual plant (personal observation). This suggests that direct anthropogenic exploitation is still ongoing. Due to its cherished blooming colors (Figure 1) around the Lunar New Year, the species has also become one of the most frequently documented wild orchids by citizen scientists. For example, there are 145 observations of *H. pumilum* on the iNaturalist platform as of May 1, 2025. This number represents approximately three‐fourths of all global observations of the genus *Holcoglossum*, which includes nine species and totals 209 observations. That is, although the genus is not commonly observed globally and new species have continued to be discovered in recent years (Fan et al. 2009; Wojtas et al. 2024), the Taiwanese *H. pumilum* is surprisingly well known and sought after during its blooming season. A conservation genetic study of this species would become a valuable resource to demonstrate current conservation efforts and help with public engagement, including the involvement of citizen scientists in combating biodiversity loss (Courchamp et al. 2018).

In this study, we applied a double‐digest restriction‐site associated DNA sequencing method (ddRADseq; Peterson et al. 2012) to generate genome‐wide single‐nucleotide polymorphism (SNP) data to study the conservation genomics of *H. pumilum* . Specifically, we collected samples from both popular forest trails and remote pristine forest habitats that required several days of hiking to access (Figure 1). These collection sites are located within four major mountain ranges of Taiwan, each with a unique geological history (Ali 2018) and potentially distinct environmental conditions (Li et al. 2013). We first analyzed the data to investigate whether population subdivision could be detected in Taiwan, either among sampling sites or across major mountain ranges. We then examined whether any observed genetic differentiation was associated with geographical distance and whether the variation of genetic diversity within populations exhibited any geographic patterns. Subsequently, we conducted genotype–environment association tests to determine whether specific genotypes or variations in allele frequencies at certain loci were linked to particular environmental variables, such as differences in annual precipitation among sampling sites. Finally, we estimated changes in effective population size over time to assess whether historical demographic fluctuations were associated with specific climatic events. In addition, we used genomic data to estimate the contemporary effective population size of the species (Do et al. 2014), which is a key parameter to inform conservation policy (Frankham et al. 2014; Hoban et al. 2020).

## 2 Materials and Methods

### 2.1 Sample Collection and Molecular Data

A total of 64 *H. pumilum* individuals were collected between 2020 and 2023 from 18 localities across four major mountain ranges in Taiwan (Figure 1). Collection permits were issued by the national parks, forest reserves, local governments, and the Forestry and Nature Conservation Agency, Ministry of Agriculture. Most orchid samples were hand‐collected from fallen branches after storms and typhoons, ensuring that healthy, thriving individuals remained undisturbed on living trees. While the collected samples were generally fresh, they were expected to perish soon due to their detachment from their microhabitats (Matelson et al. 1993). The samples were preserved in a −80°C freezer prior to DNA extraction. The specimens have been vouchered at the Herbarium, Biodiversity Research Museum, Academia Sinica, Taipei, Taiwan (HAST; Data S3). Genomic DNA was extracted using the NautiaZ Plant DNA Mini Kit (Nautia Gene) following the manufacturer's protocol. Specifically, approximately three leaf tips per sample were cut and homogenized using liquid nitrogen before DNA extraction. The extracted DNA was then assessed for concentration and quality using a Qubit dsDNA kit (Thermo Fisher).

We applied a ddRADseq (Peterson et al. 2012) method to generate a reduced‐representation genomic library using the 64 samples following protocols outlined in De Vivo et al. (2023). Briefly, DNA samples were digested using *EcoRΙ* and *MseΙ* enzymes (New England Biolabs). The processed samples were then individually barcoded with specific customized DNA sequence tags via T4 ligase (New England Biolabs), amplified with 12 cycles of PCR reaction (Phusion High‐Fidelity DNA Polymerase, New England Biolabs) to attach Illumina sequencing adaptor, and pooled for Illumina sequencing. The sequencing was performed on an Illumina NovaSeq 6000 platform by Tri‐I Biotech Inc. (Taipei, Taiwan).

### 2.2 Processing Sequencing Results

The sequence data were de novo assembled using the ipyrad pipeline (version 0.9; Eaton and Overcast 2020). Base calls with a Phred score below 33 were converted to “N”s, and reads containing more than two Ns were excluded. Within each sample, filtered reads were clustered at a 90% similarity threshold (these parameters were arbitrarily set but have been shown to work well in similar studies; c.f., De Vivo et al. 2023; Huang et al. 2024). Error rates and heterozygosity were estimated from the locus clusters for each individual. These averages were then used to generate consensus sequences, where heterozygous sites were recorded using the standard IUPAC code. Clusters with a sequencing depth below six (< 6) were discarded. Additionally, read clusters were excluded if more than two alleles were detected at any site because we assumed that *H. pumilum* is diploid. This assumption was based on the general diploidy of *Holcoglossum* orchids (Jin et al. 2007). Consensus loci built within samples were then clustered across samples using a 90% similarity threshold and aligned while allowing a maximum of six indels. Three output datasets were generated to account for different levels of missing data: one with < 20% missing data (if a locus was present in more than 51 individuals, it was retained; m51 dataset) and another with < 50% missing data (if a locus was present in more than 32 individuals, it was retained) before subsequent analyses. A third one was generated with a minimum of four individuals as default setting (m4 dataset). All three datasets were used to evaluate the impact of missing data on the estimates of population genetic summary statistics and demographic inferences. The final output files were saved in the variant call format (VCF).

### 2.3 Estimating Population Genetic Summary Statistics

We calculated multilocus heterozygosity (Coltman et al. 1999) for each individual orchid using the *MLH* function from the inbreedR package (Stoffel et al. 2016). To randomly select one SNP per locus from the filtered and assembled ddRADseq datasets, we used a customized Perl script (https://www.biostars.org/p/313701/). The edited VCF files were then imported into R using the read.vcfR function from the vcfR package (Knaus and Grünwald 2017) to estimate MLH. We also estimated nucleotide diversity (π) for each local population (pi_*L*_), as well as for all samples from the same mountain range (pi_*M*_), using the *pi.dosage* function from the *hierfstat* package. Note that for nucleotide diversity estimates, we used the original output datasets from *ipyrad* instead of the subsetted datasets that included only one SNP per locus (see previous section). To provide the total sequence length (*L*) when estimating nucleotide diversity, we multiplied the number of retained loci in the dataset by 150, which was the sequence length of our Illumina runs.

### 2.4 Estimating Population Structure

We estimated an individual‐based maximum likelihood tree for each dataset using the concatenated sequence output from the ipyrad assembly pipeline in PHYLIP format. When inferring phylogenetic relationships among individuals, missing data can be informative (Huang and Knowles 2016). Therefore, excluding loci based on the number of individuals successfully sequenced may introduce bias in phylogenetic estimation. As a result, we reconstructed the phylogeny for all three datasets to investigate whether missing data may have impacts on the grouping of individuals based on phylogenetic analysis. To reconstruct the phylogenetic tree, we used raxmlGUI 2.0 (Stamatakis 2014; Edler et al. 2020) under the raxmlHPC‐AVX mode with the GTRGAMMA model of molecular evolution. Branch support was assessed using 100 rapid bootstrap replicates.

To visualize the genetic diversity in our datasets, we first used the scaleGen function (*adegenet* package; Jombart 2008) to handle missing data by replacing them with the mean value (NA.method = “mean”). We then performed a principal component analysis (PCA) (center = TRUE, scale = TRUE) using the dudi.pca function from the *ade4* package (Dray and Dufour 2007). To statistically test for the existence of genetic groups, we used the snapclust.choose.k function from the R package *adegenet* to determine the optimal number of genetic clusters in our datasets (*K* ranges from 1 to 5), based on AIC and BIC values. The snapclust algorithm uses a likelihood‐based approach to identify the number of genetic groups that best explain the observed genetic diversity, while assigning individuals to their respective clusters. This method has been shown to accurately infer population genetic structure and provide significantly faster computational performance compared to other commonly used methods (Beugin et al. 2018). Additionally, we estimated the optimal number of ancestral populations explaining the observed genotypic diversity in our datasets using the entropy criterion (Alexander and Lange 2011; Frichot et al. 2014), implemented in the snmf function from the R package *LEA* (Frichot and François 2015). VCF files were converted to genotype format using the vcf2geno function in the R package *LEA*. For snmf analyses, we specified 100 repetitions with the number of ancestral populations ranging from 1 to 10 for cross‐validation. It is important to note that for PCA, snapclust, and snmf analyses, only the m32 and m51 datasets were used, as the methods can be sensitive to missing data. Additionally, only the subsetted datasets containing one randomly chosen SNP per locus were used for the three analyses.

Additionally, we used the RADpainter and *fineRADstructure* pipelines (version 0.3.2; Malinsky et al. 2018) to infer population structure. This approach utilizes haplotype linkage information within individual loci to construct a coancestry matrix, which helps with population inference. First, we converted the allele output file from the *ipyrad* processing into the required input format using a customized python script available at the following link (https://github.com/edgardomortiz/fineRADstructure‐tools). We then followed the authors' online protocol for data analysis (https://www.milan‐malinsky.org/fineradstructure). The results were visualized using an R script included in the *RADpainter* and fineRADstructure program. Analyses were conducted only on the m32 and m51 datasets, as > 50% missing data may have impacts on the resulting coancestry matrix.

To assess the impact of geographical distance on genetic differentiation among populations, we conducted isolation by distance (IBD) analyses. Genetic distances between population pairs were estimated using the dist.genpop function from the *adegenet* package. Given the various ways genetic distance can be measured, we reported all five types of genetic distances available in the function. Specifically, the Mantel test for IBD has been criticized for the lack of power to detect underlying patterns, and the power to detect such patterns may be affected by the transformation of distance matrices (Quilodrán et al. 2025). Euclidean distances between populations from different geographical localities (Figure 1) were calculated using the dist function. Mantel tests were then performed to evaluate the statistical significance of the association between geographical and genetic distances. Specifically, we used the mantel.randtest function from the *ade4* package to conduct Mantel tests with 999 permutations. As with previous analyses, IBD tests were performed only on the m32 and m51 datasets.

Finally, to further evaluate the association between genetic variation and geographical distance, we performed Procrustes analyses (e.g., Huang et al. 2024). The first two principal components (PC1 and PC2) from the previous analyses were compared with the geographical coordinates (latitude and longitude) of the samples using the protest function from the vegan package (Dixon 2003). Procrustes analysis minimizes the sum of squared Euclidean distances between the genetic PCA map and the corresponding geographic map of sampling locations while preserving relative pairwise distances within each dataset. This procedure produces an optimal transformation that maximizes the congruence between genetic and geographical structures. Statistical significance was assessed through 10,000 permutations.

### 2.5 Genotype–Environment Association

We used redundancy analysis (RDA) to assess genotype–environment associations (GEA) based on genomic datasets (m32 and m51) and bioclimatic variables from WorldClim. RDA detects groups of loci whose allele frequencies covary with multivariate environmental variables (Rellstab et al. 2015; Capblancq et al. 2018; Forester et al. 2018). We obtained 19 bioclimatic variables at a 2.5‐arc‐minute spatial resolution using the worldclim_global function from the *geodata* package (Hijmans et al. 2025) and extracted climatic data for each individual using geographical coordinates and the extract function from the *raster* package (Hijmans 2025). Before conducting RDA, we checked for correlations among the 19 bioclimatic variables and removed one from each highly correlated pair (absolute *r* > 0.7), as identified using the pairs.panels function from the *psych* package (Revelle 2025). Four bioclimatic variables that showed minimal collinearity (see Results) were retained for GEA analyses. RDA was performed using the rda function in the *vegan* package (Dixon 2003). To evaluate how much genetic variation was explained by constrained ordination, we used the RsquareAdj function in *vegan*. The statistical significance of the RDA model, both the full model and individual constrained axes, was assessed with the anova.cca function using 999 permutations. Additionally, we examined variance inflation factors for the four included bioclimatic environmental variables using the vif.cca function in *vegan*. Finally, potential outlier loci showing significant associations (> 3SD) with environmental variation were identified and reported along with their best bioclimatic predictor variable. Our RDA analyses in general followed an online tutorial (https://popgen.nescent.org/2018‐03‐27_RDA_GEA.html). Note that because our results did not reveal a clear correlation between genetic data and geographical distance (see Results section), we did not account for spatial genetic structure in the GEA analyses.

### 2.6 Estimating Effective Population Size

We used *Stairway Plot 2* (version 2.1.1; Liu and Fu 2020) to estimate changes in effective population size over historical time without assuming specific historical events. For this analysis, we applied a mutation rate of 10^−9^ per site per generation and a generation time of 5 years (c.f. Leal et al. 2025). We first generated a folded site frequency spectrum (SFS) file using the *easySFS* script (https://github.com/isaacovercast/easySFS), which converts VCF files obtained from the *ipyrad* pipeline. Since the *ipyrad* outputted VCF file contained only variable sites, we provided the total number of sequenced sites based on the number of loci from the *ipyrad* output. This information is crucial for *Stairway Plot 2*, as it requires the total count of sequenced sites (L), including both variable and invariable sites. Additionally, *easySFS* accounts for missing data and constructs SFS files by projecting them across different numbers of haploid sequences. For the *Stairway Plot 2* analysis, we used seven randomly selected breakpoints and generated 1000 inputs (ninputs). To assess the robustness of our results, we performed 200 bootstrap replicates to estimate confidence intervals for inferred effective population sizes over time. The analyses were performed for all three datasets with different levels of missing data percentage.

Additionally, we estimated the current effective population size using contemporary samples implemented in *NeEstimator v2* (Do et al. 2014). Since *Stairway Plot 2* may lack sufficient resolution to detect recent demographic changes and may not provide an accurate estimate of the current effective population size, we chose to apply this alternative approach. Specifically, current effective population size is a critical parameter for conservation planning. We first converted the filtered one‐SNP‐per‐locus files (from m32 and m51 datasets) from VCF format to GenePop format using *PGDSpider* (Lischer and Excoffier 2012). The converted GenePop files were then imported into *NeEstimator v2*, where we estimated the current effective population size using the linkage disequilibrium model under the assumption of random mating.

## 3 Results

### 3.1 Summary of Sequencing and Assembling Results

A total of 48,085,995 single‐end raw reads, each 150 bp long, were generated from the 64 samples. On average, each individual received 751,344 reads, with a standard deviation of 394,081. The highest number of reads for an individual was 1,919,110, while the lowest was 202,722. After processing with ipyrad, a total of 63,949 de novo loci were assembled. However, only 31,445, 1064, and 231 loci were retained for the m4, m32, and m51 datasets, respectively. In the m4 dataset, each individual was represented in an average of 5639 loci, with a standard deviation of 4581. In the m32 dataset, the mean number of loci per individual was 696, with a standard deviation of 233. Finally, in the m51 dataset, individuals retained an average of 212 loci, with a standard deviation of 17. Details about the sequencing and assembling results can be found in Data S2; Figure S1.

### 3.2 Population Genetic Summary Statistics

The estimated multilocus heterozygosity (MLH) using the m4 dataset ranges from 0.059% to 0.164%, with a mean of 0.104% and a standard deviation of 0.026% (Data S2). In the m32 dataset, MLH ranged from 0.026% to 0.117%, with a mean of 0.071% and a standard deviation of 0.025% (Figure 1). For the m51 dataset, six samples exhibited no heterozygosity across the 231 retained loci (MLH = 0). The average MLH for the m51 dataset was 0.034%, with a standard deviation of 0.034% and a maximum value of 0.167% (Figure 1). Note that missing data may have affected the estimated MLH values in the m4 and m32 datasets, but not in the m51 dataset; specifically, a statistically significant correlation between the number of retained loci and the estimated MLH values was detected in the m4 and m32 datasets (*p* = 4.548 × 10^−16^ and 1.482 × 10^−12^, respectively, based on linear regression; Figure S2). Additionally, the estimated MLH values for each individual were statistically significantly correlated among datasets (*p* < 0.01 based on linear regression), except between the m4 and m51 datasets (*p* = 0.905; Figure S3). Interestingly, we detected a statistically significant latitudinal gradient in MLH values in the m32 and m51 datasets (*p* = 4.5 × 10^−6^ and 0.001, respectively, based on linear regression), with samples from southern localities showing higher heterozygosity than those from northern Taiwan (Figures 1 and S4).

Similarly, our results demonstrated a decrease in genetic diversity, π, within populations along the latitudinal gradient (Figure 1). Specifically, local populations from the Southern Central Mountain Range (SMR) and Alishan Mountain (ALS) often exhibit higher π values than those from the Central Mountain Range (CMR) and Xueshan Mountain (XSM). As a result, we found that π is highest in SMR, followed by ALS, whereas the lowest π value was detected in XSM. This pattern of geographic variation in genetic diversity across local populations and among mountain ranges is consistent between the m32 and m51 datasets. Note that some local populations have a π value of 0 (denoted as NA in Data S2), which is due to the presence of only a single sequenced individual from those populations (Figure 1).

### 3.3 Population Structure

The reconstructed ML phylogenies based on different datasets showed weak support for any grouping of individuals (Figures S5–S7). Although some groupings were present, their composition varied across datasets, and bootstrap supports were generally below 10. Most of the genetic diversity was found within individuals, as branches leading to individual samples were typically longer than those connecting different lineages. However, this pattern was less apparent in the phylogeny reconstructed using the m51 dataset, where many individuals shared highly similar genotypes (Figure S7).

**FIGURE 2:**
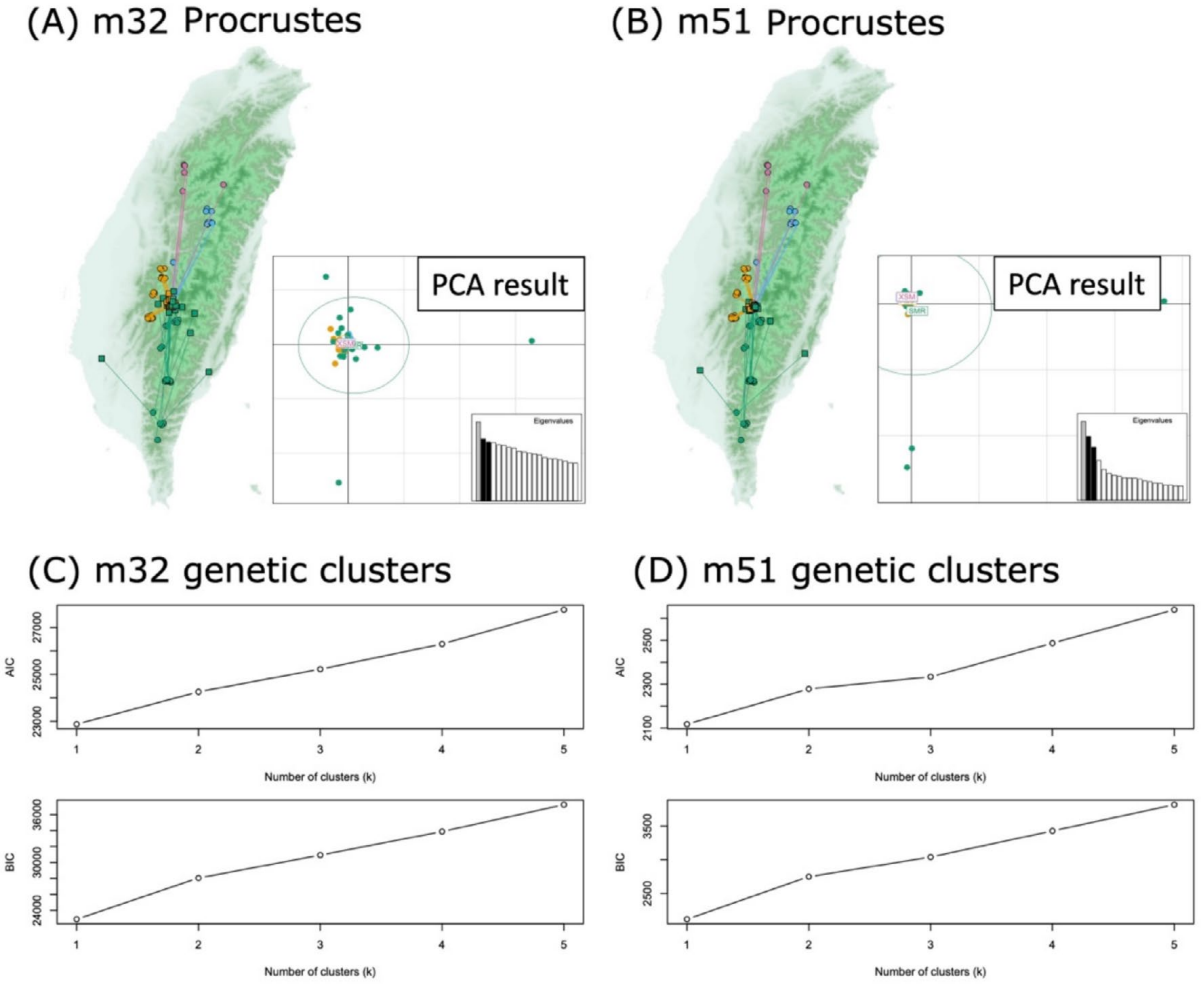
Results of genetic grouping and geographical association of genetic variation using ddRADseq datasets. Results from Procrustes analyses based on two different filtering strategies (m32 and m51) of the ddRADseq dataset are shown in panels (A) and (B). Population color schemes match those in Figure 1, representing their geographical origins across major mountain ranges in Taiwan. Colored filled squares represent the PC coordinates of the genetic data, whereas circles indicate the geographical origins of the samples. Straight lines connect each genetic data point to the geographical origin of the corresponding individual. Principal component analysis (PCA) results used for the Procrustes analyses are embedded in the lower right corners of panels (A) and (B). The best estimates of the number of genetic clusters, determined using AIC and BIC for the two datasets, are shown in panels (C) and (D). All results indicate no clear geographical genetic grouping in our study system.

Similarly, we did not see any genetic clustering of individuals based on visual inspection of the PCA results (Figure 2). Genotypes from different mountain ranges occupied overlapping regions in PCA space for both the m32 and m51 datasets. However, samples from SMR spanned a much broader PCA space than those from other populations, followed by samples from ALS. Statistically, the optimal number of genetic groups explaining the observed genetic diversity in both m32 and m51 datasets was one, based on both AIC and BIC values from the *snapclust* analyses (Figure 2). Likewise, the best number of ancestral populations needed to explain the observed genetic variation was one, as indicated by using the entropy criterion and cross‐validation results, regardless of the input dataset (Figure S8). Our coancestry plots also indicated that individuals from both the same and different populations shared similar amounts of alleles (Figures S9 and S10), which suggests no clear genetic grouping. However, based on the m32 dataset, there were a few exceptions: two individuals from MF and three from ZZG shared slightly more alleles with each other than with individuals from other populations (Figure S9). This pattern was not observed in the m51 dataset (Figure S10).

**FIGURE 3:**
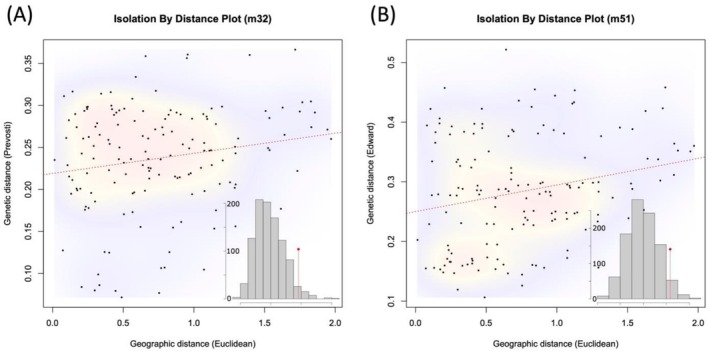
Tests for genetic isolation by geographical distance (IBD) were conducted between pairs of populations using the m32 (A) and m51 (B) datasets. Statistically significant IBD was detected in both datasets; however, significance was only observed when different methods for calculating genetic distance between populations were applied. Specifically, in the m32 dataset, significant IBD was found when genetic distance was estimated using Prevosti's method (method = 5), whereas in the m51 dataset, significant IBD was detected using Edward's distance calculation (method = 2). A red dashed line in the scatter plot indicates the estimated linear regression between geographical and genetic distances. A background color patch depicts the density of points in the scatterplots; a warmer color (e.g., red) indicates a higher density, whereas a colder color (e.g., purple) indicates a lower density. Mantel test results with 1000 permutations (frequencies shown at the y‐axis) for the significant comparisons are shown in the bottom‐right corner of the figures. The empirical data are represented by a solid red line.

Finally, we detected traces of isolation by distance (IBD) in both the m32 and m51 datasets. However, IBD was only evident when genetic distances between populations were calculated using specific methods (Figure 3). In the m32 dataset, statistically significant IBD was detected via the Mantel test only when Prevosti's distance (method = 5) was used to represent genetic distances. In contrast, in the m51 dataset, statistical support for IBD was found only when genetic distances were calculated using Edwards' distance (method = 2). All other methods of calculating genetic distances resulted in non‐significant IBD for both datasets. Similarly, we only detected marginal statistical significance of geographical association of genetic variation based on Procrustes analyses (Figure 2). In the m32 dataset, a *p* value of 0.0525 was estimated between genetic distance and geographical distance (Procrustes sum of squares = 0.9521; correlation in a symmetric Procrustes rotation = 0.2189). In the m51 dataset, a *p*‐value of 0.1379 was estimated between genetic distance and geographical distance (Procrustes sum of squares = 0.9638; correlation in a symmetric Procrustes rotation = 0.1901).

### 3.4 Genotype–Environment Association

Four bioclimatic variables with absolute Pearson's correlation coefficients below 0.7 were retained for GEA analyses (Figure S11). These variables were BIO3: Isothermality, BIO7: Temperature Annual Range, BIO11: Mean Temperature of the Coldest Quarter, and BIO12: Annual Precipitation. Redundancy analysis (RDA) using these environmental predictors explained 1.13% and 2.28% of the total genetic variation in the m32 and m51 datasets, respectively. The RDA results were statistically significant for both datasets (*F* = 1.1806, *p* = 0.007 for m32; *F* = 1.3324, *p* = 0.008 for m51). In the m32 dataset, the first RDA axis explained 38.59% of the constrained variation and was statistically significant, whereas the second axis explained 27.89% but was only marginally significant (*p* = 0.053). In the m51 dataset, the first axis explained the majority of the variation (59.48%) and was the only axis with statistical significance. For both datasets, the Variance Inflation Factors (VIFs) for the four predictor variables were below 5, which suggests that multicollinearity was not an issue in the models.

**FIGURE 4:**
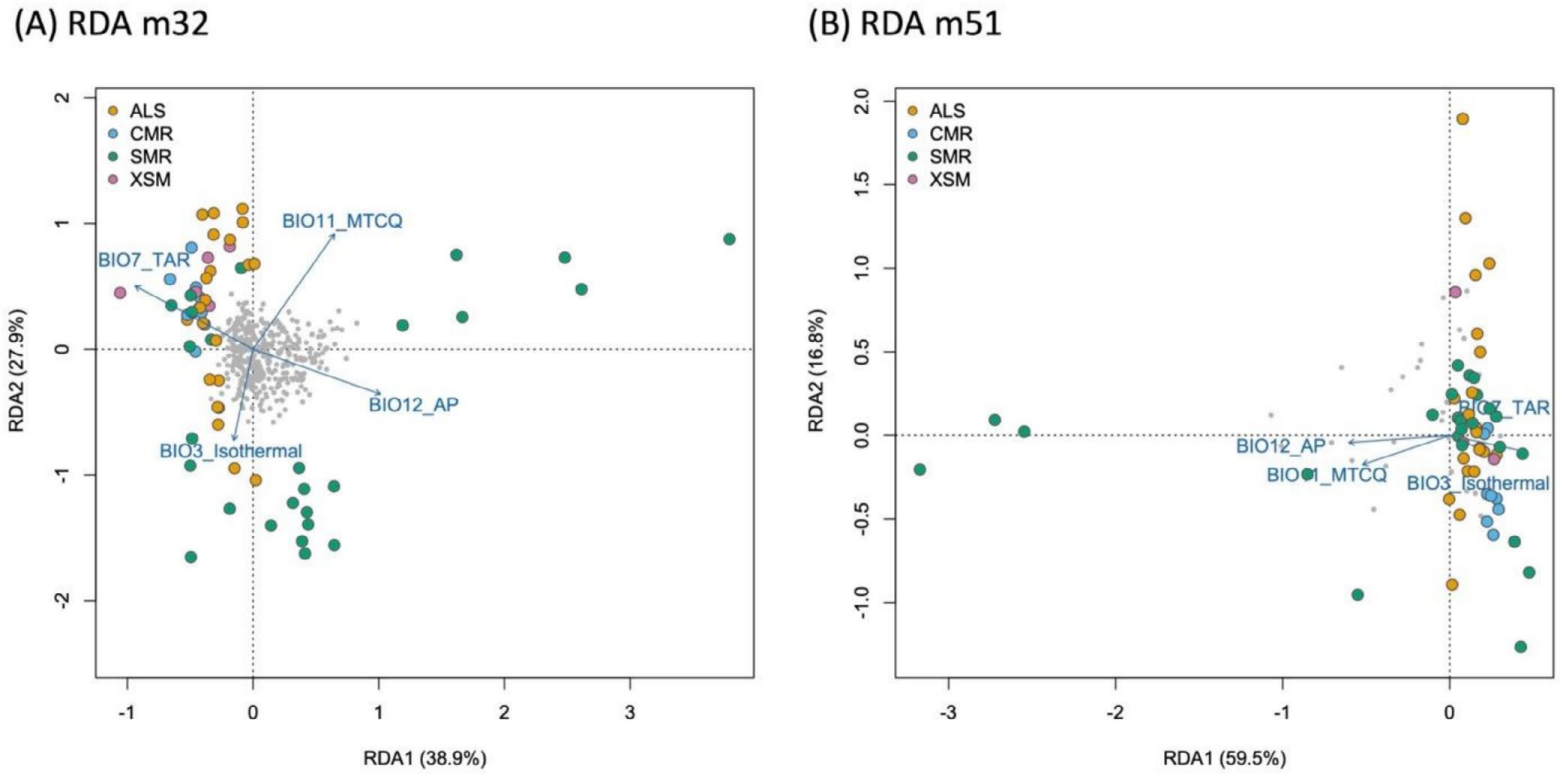
Results of genotype–environment association analysis using redundancy analysis (RDA) with two different datasets that applied different filtering strategies. Gray dots represent the distribution of SNPs in the two‐dimensional RDA space, while colored dots indicate individual genotypes from different samples, with colors corresponding to their mountain range of origin (as shown in Figure 1). In both datasets, RDA was statistically significant; however, environmental variables explained only a small proportion of genetic variation (1.13% for the m32 dataset and 2.28% for the m51 dataset). Specifically, in the m32 dataset, only RDA1 was statistically significant, whereas RDA2 was marginally significant (*p* = 0.053). In the m51 dataset, only RDA1 was statistically significant.

Similar to the PCA results, individual genotypes from SMR occupied a broader space in the RDA biplots, followed by genotypes from ALS (Figure 4). However, no distinct clustering patterns were observed based on the different environmental predictors. Using the m32 dataset, we identified five putative outlier loci based on a threshold of three standard deviations (Figure S12); all five loci were most strongly associated with BIO12: Annual Precipitation. No outlier loci were detected in the m51 dataset using the same criterion. Note that the five loci detected as outliers in the m32 dataset were not retained in the m51 dataset due to the filtering of missing data percentage.

### 3.5 Historical and Contemporary Effective Population Size

**FIGURE 5:**
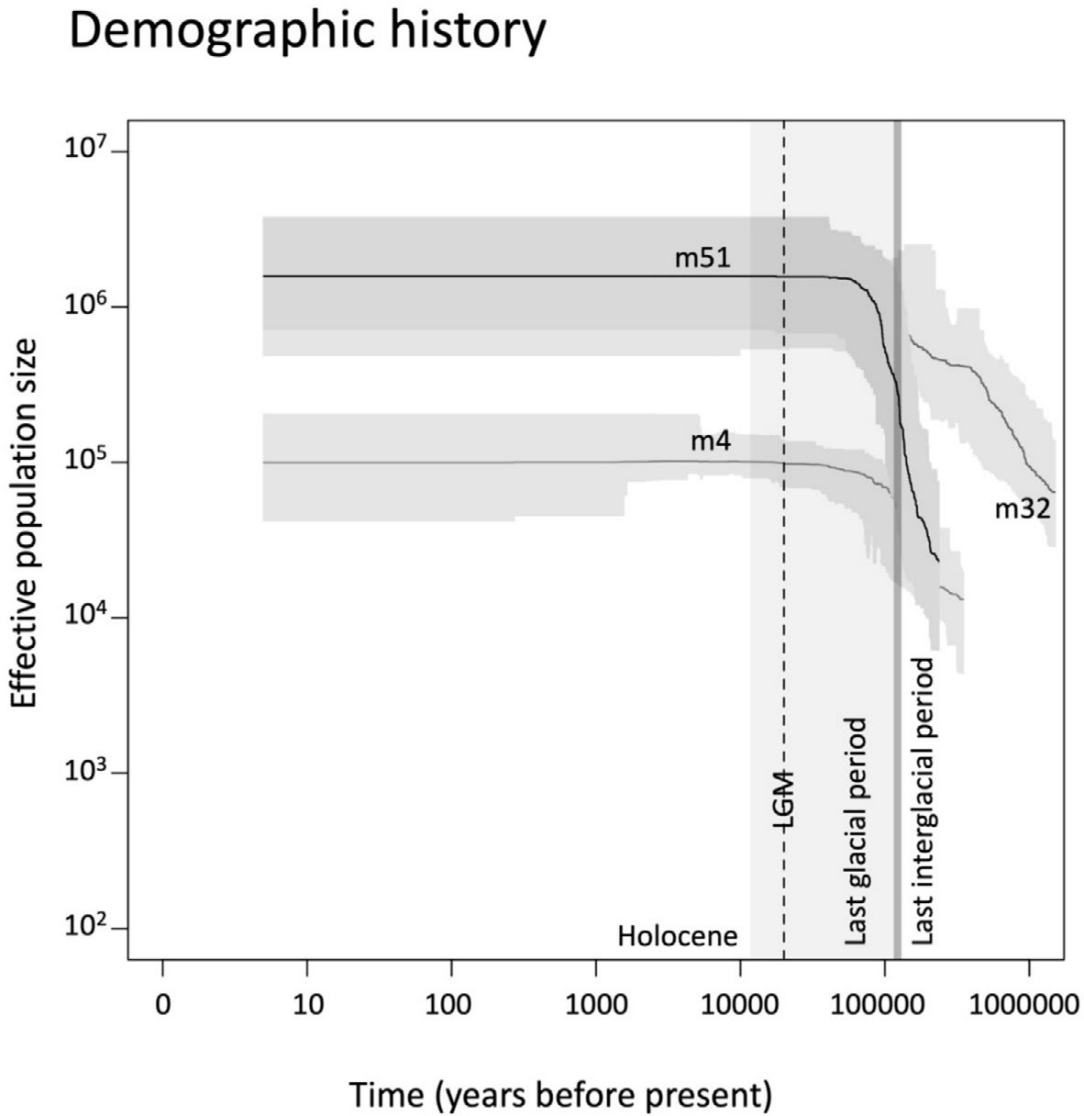
Reconstructed demographic history of *H. pumilum* using Stairway Plot 2. Note that both the estimated effective population sizes and the time before the present are shown on a log scale. Demographic histories were estimated from folded site frequency spectra (SFS) derived from three different filtering strategies (m4, m32, and m51). Solid lines represent the mean estimated effective population sizes over time, whereas the shaded gray areas indicate the 95% confidence intervals. Key geohistorical periods are highlighted: The last glacial period in light gray, the last glacial maximum (LGM) with a vertical dashed line, and the last interglacial period with a dark gray rectangle. The Holocene is indicated with a white background. Note that the estimates for the m32 and m51 datasets overlapped in more recent times.

Our results indicate that the effective population size of *H. pumilum* began to increase prior to the last interglacial period and continued to rise during the last glacial period. Subsequently, the population size plateaued during the last glacial period, but before the Last Glacial Maximum (LGM) (Figure 5). This demographic trend was consistent across all three datasets, each of which used different data filtering strategies. Interestingly, the dataset allowing the most missing data (m4) inferred the smallest effective population size, while the dataset with the least missing data (m51) inferred the largest. Meanwhile, the m32 dataset was able to reconstruct demographic history extending the farthest back in time (Figure 5).

Using the linkage disequilibrium method implemented in NeEstimator v2, the estimated contemporary effective population size based on the m32 dataset, without applying a minimum allele frequency threshold, was 179.3, with a 95% confidence interval (CI) ranging from 158.5 to 206.0. Similar estimates were obtained when different minimum allele frequency thresholds were applied and when singleton allele loci were excluded from the analysis (Table 1). In contrast, the m51 dataset yielded a much smaller effective population size. Specifically, without applying any allele frequency threshold, the estimated effective population size was 12.1, with a 95% CI ranging from 11.0 to 13.4. These estimates remained similar when different allele frequency thresholds were used and when loci with only singleton alleles were excluded (Table 1).

**TABLE 1:** Estimated contemporary effective population sizes (NeEstimator v2).

	0^a^	0.01^a^	0.02^a^	No singleton
m32	179.3 (158.5–206.0)^b^	560.9 (388.2–1001.3)^b^	274.5 (201.0–428.5)^b^	187.6 (149.1–251.2)^b^
m51	12.1 (11.0–13.4)^b^	22.3 (16.9–30.0)^b^	55.6 (22.9–807.4)^b^	20.0 (14.6–27.8)^b^

^a^ Results applying different thresholds of the lowest allele frequency.

^b^ Numbers in parentheses represent the 95% confidence interval range.

## 4 Discussion

Our genomic estimates of genetic diversity revealed a clear latitudinal gradient, where higher heterozygosity and nucleotide diversity were observed in southern populations (Figures 1 and S4). Although we did not detect genetic clustering (Figures 2 and S8) and we only found limited evidence of isolation by distance (Figure 3), southern populations occupied a broader PCA space, supporting the results that these populations harbor higher genetic diversity. Our results also showed significant genotype–environment associations, which were primarily linked to annual precipitation (Figure 4). Similarly, the southern populations tend to harbor more diverse genotypes that occupy a broader environmental space than the northern ones (cf. Figures 2 and 4). Results from demographic reconstructions suggested a historical population expansion before the Last Glacial Maximum (Figure 5), while contemporary Ne estimates varied substantially depending on filtering strategies (Table 1); specifically, the estimated contemporary Ne ranged from around 12 (< 50) to 560 (> 500) individuals, but most estimates of Ne were smaller than 500. In the following sections, we discuss the factors that may have contributed to the origin and maintenance of genetic diversity in *H. pumilum* in Taiwan, and how this genetic information can inform conservation strategies to help with the long‐term survival of the species.

### 4.1 The Impact of Missing Data on Population Genomic Inferences

Our results demonstrate that varying levels of missing data had apparent yet uneven impacts on different population genetic summary statistics. The most affected estimates were multilocus heterozygosity (MLH) and contemporary effective population size (Ne). Specifically, MLH values were significantly correlated with the number of retained loci in the m4 and m32 datasets but not in the most stringent dataset (m51). Similarly, the estimated Ne values differed substantially between datasets, with the m32 dataset (allowing more missing data) yielding larger estimates and the m51 dataset (least missing data) producing smaller ones. In contrast, measures of genetic diversity within populations (π), the lack of population genetic structure (as indicated by phylogenetic, PCA, and clustering analyses), and the genotype–environment association (GEA) results were more robust to missing data. The detection of isolation by distance (IBD) was only marginally sensitive to dataset choice and the distance metric used. Our results thus unravel that summary statistics capturing individual heterozygosity and demographic parameters, those most relevant to conservation inferences, were particularly sensitive to missing data. In contrast, estimates commonly used in phylogeographic and landscape genetic studies, including genetic structure and genotype–environment associations, remained relatively stable across different filtering thresholds.

As mentioned in the previous paragraph, both MLH and estimates of Ne were highly sensitive to the stringency of missing‐data filtering, which demonstrates the critical role of locus retention in conservation genetic inferences. For MLH, relaxing missing‐data thresholds may retain more variable loci that would be excluded under more conservative filtering strategies, thereby increasing the odds of capturing heterozygous sites. Empirically, it has been shown that allowing higher levels of missing data does retain more loci and potentially enhances the statistical power for heterozygosity estimation (Chattopadhyay et al. 2014). Similarly, a study based on simulations has demonstrated that relaxed missing‐data thresholds can yield higher observed heterozygosity compared to more stringent cut‐offs (Schmidt et al. 2021). Furthermore, Ne estimates were also influenced by filtering stringency. A more stringent strategy tends to include fewer polymorphisms, whereas more permissive filters introduce loci with higher missingness or higher variability. As a result, a more stringent filtering strategy may lead to a smaller estimate of Ne. This pattern has been observed in empirical studies, including RAD‐seq analyses of thornback rays (Marandel et al. 2020) and conservation‐genomics studies in plants (Gargiulo et al. 2024). Our results thus support the importance of carefully considering missing‐data filtering strategies in conservation genetic studies because different thresholds may lead to different estimates for key parameters. Reconciling these differences requires an integrative approach that explicitly compares results across multiple filtering strategies rather than relying on a single, arbitrarily chosen threshold. For conservation planning in *H. pumilum* , we may want to place a greater weight on heterozygosity estimates derived from moderately relaxed filters, as these are likely to better reflect genetic diversity within populations. On the other hand, Ne estimates derived under a stringent filtering strategy should be interpreted as a conservative minimum.

### 4.2 Latitudinal Genetic Diversity Gradient and the Absence of Population Subdivision

Our genomic analysis of *H. pumilum* reveals neglectable population structure and subtle geographic patterns of genetic variation across its range in Taiwan. Specifically, despite extensive sampling across diverse habitats and multiple mountain ranges, we found that there is no strong evidence of distinct genetic clusters among populations. Phylogenetic, PCA, and genetic clustering analyses consistently indicated no genetic differentiation among populations. While some evidence of isolation by distance was detected, it was method‐dependent and not robust across all methods of measuring genetic distance. Note that the lack of IBD detected using the Mantel test should be taken with caution because there may be an issue related to the limited power of the test to detect such a pattern (Quilodrán et al. 2025). Nevertheless, we did not detect a significant association between the geographical origins of the samples and their genetic differences in a two‐dimensional PC space, based on the results of the Procrustes analyses. All these findings mentioned above suggest that *H. pumilum* likely maintains a relatively panmictic genetic structure across its range with subtle geographic signals in genetic diversity, which implies extensive gene flow or a recent population expansion. Note that a recent study using similar genomic methods identified statistically significant population subdivision in an endemic terrestrial orchid in Taiwan (Chao et al. 2021); however, epiphytic orchids have been shown to exhibit less apparent genetic structure than their terrestrial counterparts in general (Phillips et al. 2012). For example, it has been hypothesized that epiphytic orchids often exhibit low genetic structure because their tiny wind‐dispersed seeds and mobile pollinators promote high gene flow across populations (Alcantra et al. 2006; Zhang et al. 2019).

An intriguing pattern observed in the geographical distribution of *H. pumila* is that it can only be found on the western part, or the windward side of the southwest monsoon (Shen, Chou, et al. 2025), of the Central Mountain Range of Taiwan (Figure 1). Given that the geographic range of the genus *Holcoglossum* extends from South Indochina to Taiwan (Zhao et al. 2020), and that its seeds are primarily wind‐dispersed (Xiang et al. 2012; Lin and Liu 2025), it is not surprising that *H. pumilum* , which is the easternmost distributed species in the genus, shows a geographically constrained distribution in Taiwan, particularly limited by the Central Mountain Range. Specifically, the Central Mountain Range consists of more than 100 peaks over 3000 m above sea level, which far exceeds the known altitudinal distribution limit of the species (< 2500 m; Lin and Liu 2025). Note that the blooming season of *H. pumilum* occurs during the winter, from late October to late February, when the dominant monsoon system is the northeast monsoon. However, the capsules of the orchid mature in summer, and the seeds are therefore likely released and dispersed during the summer, when the southwest monsoon becomes the prevailing monsoon system in Taiwan.

Additionally, because of the prevailing wind direction of the southwest monsoon and the geographic range of the genus, it is likely that the northern populations of *H. pumilum* are peripheral populations derived from southern populations, which may represent the genetic source of the species. As a result, we identified a statistically significant latitudinal gradient in genetic diversity, while no clear genetic breaks or population subdivision were detected in this study system (Figures 1, 2, 3). In addition, our estimated time of demographic expansion (Figure 5) corroborates with findings from a recent biogeographic study of the genus *Holcoglossum* (Zhao et al. 2020), where the authors hypothesized that changes in the global monsoon system during the Quaternary period likely facilitated the geographical expansion, dispersal, and diversification of the genus. As a result, our study further shows the critical role of seasonal monsoons in shaping intraspecific latitudinal genetic diversity gradients, a pattern that has rarely been demonstrated in empirical systems (Shen, Chou, et al. 2025).

Furthermore, despite the overall genetic homogeneity across populations (Figures 2 and 3), we detected statistically significant associations between genetic variation and changes in the values of environmental factors, particularly annual precipitation (BIO12; Figure 4). Specifically, our results from RDA revealed that a small proportion of total genetic variance could be explained by our selected bioclimatic variables, where BIO12 was the most impactful. Note that it is expected that only a small proportion of the total genetic variation would be explained by the environmental variables analyzed in our study because the majority of genetic variants obtained through our ddRADseq approach likely represent neutral variation (Sunde et al. 2022). We also found that genotypes from southern regions, especially those from SMR and ALS, not only exhibited higher genetic diversity (Figure 1) but also occupied broader multivariate space in environmental association analyses. However, we did not identify distinct genetic clusters that are associated with environmental variables, and only a few outlier loci associated with precipitation were detected. The detection of outlier loci was also subject to the impact of missing data. Nevertheless, our results unravel the importance of the southern populations of *H. pumila* , which may disproportionately harbor high genetic, and potentially adaptive, diversity.

### 4.3 Implications for Species Conservation and Opportunities for Community Engagement

Our findings provide critical information into the genetic diversity and evolutionary potential of *H. pumilum* , a miniature epiphytic orchid endemic to the island of Taiwan. The relatively small contemporary effective population sizes estimated, especially under more stringent filtering criteria (Table 1), raise concerns about the long‐term survival of the species under ongoing environmental change. Specifically, the estimates of the contemporary effective population size varied widely depending on dataset filtering strategies, with values ranging from around 12 to 560. These findings raise conservation concerns because of the small Ne estimates from the most conservative dataset (Ne < 50; cf. Frankham et al. 2014). That is, even though the species is listed as Least Concern (LC) in the Red List of Taiwanese flora, the contemporary *Ne* is likely too small to maintain adaptive genetic variants necessary for future evolvability (Ne < 500 in most estimates; Table 1). From a conservation perspective, maintaining population connectivity, protecting habitat heterogeneity, and prioritizing the conservation of high‐diversity regions like the SMR and ALS will be crucial. These areas may serve as reservoirs of genetic, and potentially adaptive, variation (Moritz 2002; Hoffmann et al. 2017).

Our genomic data also provide critical information to guide future reintroduction or assisted gene flow programs for *H. pumilum* . The observed panmictic population genetic structure indicates a high level of gene flow across regions, which implies that moving individuals between populations is unlikely to disrupt the local gene pool. As a result, the risk of outbreeding depression (Kalls et al. 2020), a common concern in conservation genetics, may be reduced or effectively managed in the *H. pumilum* system if reintroduction or genetic rescue is pursued. In addition, as mentioned above, southern populations, especially those from SMR and ALS, contain higher genetic diversity, which may include potentially adaptive variation, making them promising candidates as source populations. These genetically diverse populations could be especially helpful in restoring populations that have experienced recent reductions in census size or have disappeared from parts of their historical range. To maximize the adaptive potential and resilience of restored or reintroduced populations, we also recommend sourcing individuals from multiple genetically diverse populations (Halford et al. 2025). Specifically, it is often challenging to predict how genetic diversity will change after reintroduction and how individual genotypes may perform over time. Therefore, we argue that special emphasis should be placed on individuals from populations with higher heterozygosity and broader associations between genotype and environment, while we should also make sure that no single source population is overrepresented. In addition, matching environmental conditions between source and recipient sites, including both current and projected future climates (Sun et al. 2024), such as precipitation patterns may also help improve the odds of successful establishment and long‐term survival of reintroduced populations. Where feasible, we believe that genomic monitoring should be employed after reintroduction to assess demographic trends, the retention of genetic diversity, and the emergence of new genotypes that may be associated with local environmental condition over time (e.g., Halford et al. 2025; Forsdick et al. 2025).

Orchid conservation faces challenges in the Anthropocene, where overcollection, habitat loss, and climate change have threatened wild populations. On the other hand, due to their popularity, wild orchid conservation projects often attract public interest and can help raise awareness about challenges in biodiversity research in the Anthropocene. Our study results provide an opportunity to engage the public and promote broader participation in conservation and sustainability efforts. Population genomic approaches, as demonstrated in our study, provide essential data to assess genetic diversity, effective population size, and adaptive potential for an endemic epiphytic orchid in Taiwan. These population genetic parameters are critical for identifying conservation priorities and informing strategies (Hoban et al. 2020). In *H. pumilum* *specifically*, for example, the detection of small contemporary effective population sizes, low genetic structure, and a significant genotype–environment association point to the importance of preserving populations with high genetic diversity and evolutionary potential while maintaining habitat connectivity (Baiotto and Guzman 2025). More broadly, population genomics allows us, the practitioners of biodiversity conservation, to shift toward evolutionarily informed conservation planning by revealing fine‐scale population processes, such as locally adaptive genotypes and gene flows, that conventional methods may overlook (van Oosterhout 2024). As biodiversity loss accelerates, our study supports the idea that integrating genomic data into conservation planning is critical, and may become essential a priori resources, to support long‐term species persistence.

## Author Contributions

**Wei‐Yun Chen:** data curation (equal), methodology (equal), project administration (equal), validation (equal), writing – review and editing (equal). **Yi Sun:** data curation (equal), investigation (equal), resources (equal), validation (equal), writing – review and editing (equal). **Shu‐Ping Wu:** data curation (equal), investigation (equal), resources (equal), writing – review and editing (equal). **Jen‐Pan Huang:** conceptualization (lead), data curation (equal), formal analysis (lead), funding acquisition (lead), methodology (equal), visualization (lead), writing – original draft (lead), writing – review and editing (equal).

## Funding

The study was supported by an internal institutional fund from the Biodiversity Research Center, Academia Sinica and the National Science and Technology Council (114‐2621‐B‐001‐006‐MY3) to J‐P Huang.

## Conflicts of Interest

The authors declare no conflicts of interest.

## Supporting information


**Data S1:** ece372920‐sup‐0001‐Figures.pdf.

**Data S2:** ece372920‐sup‐0002‐Supinfo1.csv.

**Data S3:** ece372920‐sup‐0003‐Supinfo2.xlsx.

## Data Availability

The raw sequence reads have been deposited in the NCBI Sequence Read Archive (SRA) under BioProject PRJNA1269268. Accession numbers for each individual are provided in the Data S2.
