# Supplementary material for: Genomic Evidence of Low Contemporary Effective Population Size and Southern Genetic Reservoirs in an Island Endemic Epiphytic Orchid of Taiwan
Source: Ecol Evol. 2026 Jan 14;16(1):e72920. doi: 10.1002/ece3.72920 (PMC12802410; doi:10.1002/ece3.72920)
Supplement: Supplementary file 1 — Data S1: ece372920‐sup‐0001‐Figures.pdf. [file ECE3-16-e72920-s002.pdf]

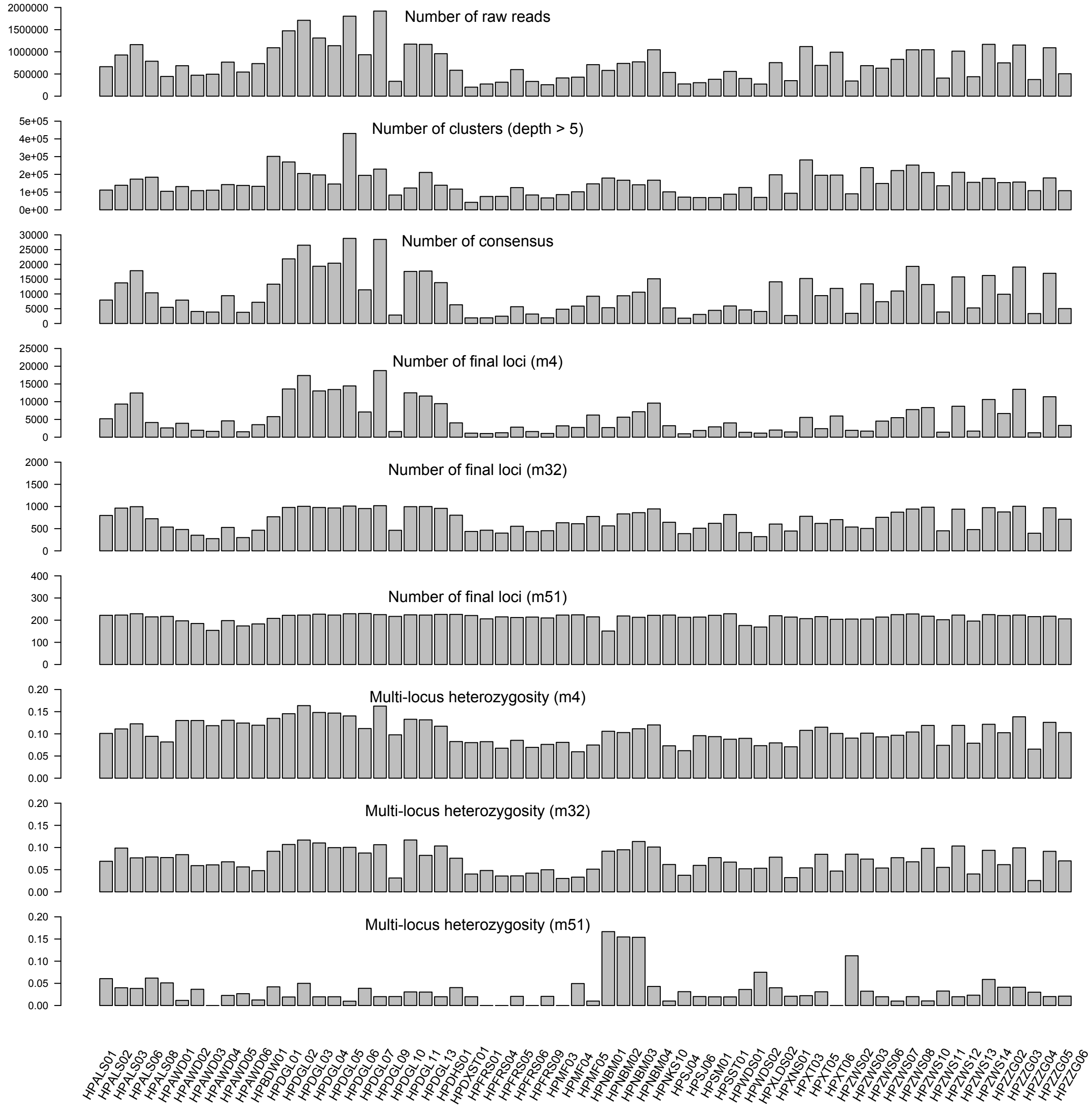

Fig. S1. Summary of the ddRADseq data after ipyrad processing.

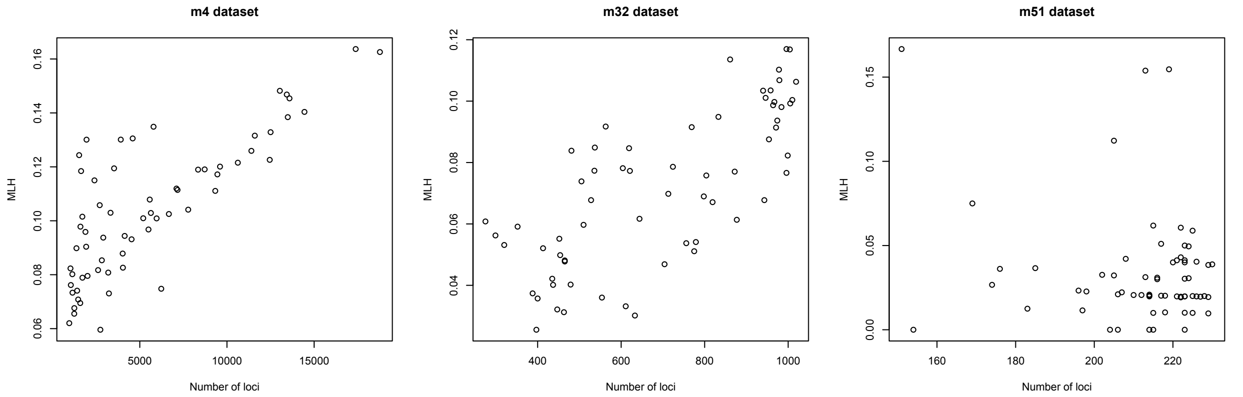

Fig. S2. Scatter plots showing the association between the numbers of retained loci and the estimated values of multi-locus heterozygosity (MLH). Statistical significant correlation was detected in m4 (P = 4.548*10^{-15}, r^2 = 0.6256, F = 106.3) and m32 datasets (P = 1.482*10^{-12}, r^2 = 0.5496, F = 77.89); however, the estimated MLH was not correlated to the number of loci in the m51 dataset (P = 0.11, r^2 = 0.026, F = 2.705).

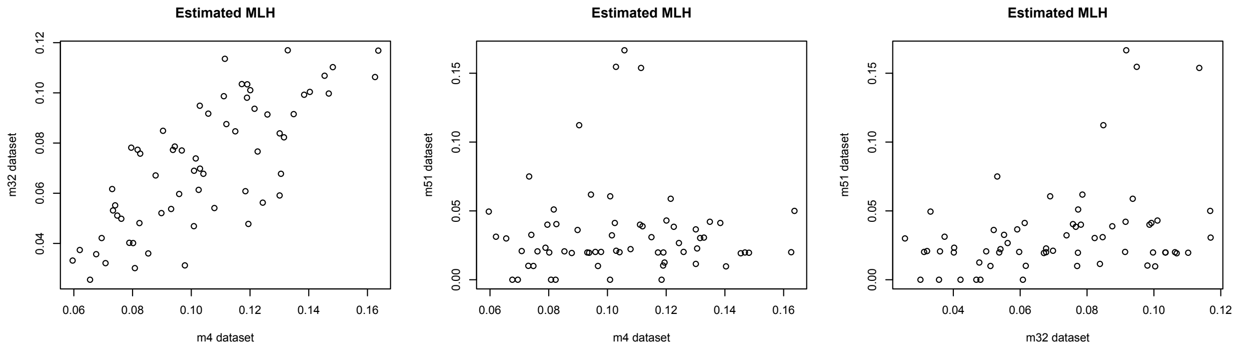

Fig. S3. Scatter plots showing the correlation between estimated values of multi-locus heterozygosity (MLH) using different datasets. Statistical significant correlations were detected between m4 and m32 datasets (P = 4.102*10^{-13}, r^2 = 0.5677, F = 83.74) as well as between m32 and m51 datasets (P = 0.0064, r^2 = 0.098, F = 7.828). While the comparison between m4 and m51 datasets was statistically insignificant (P = 0.9054, r^2 = -0.0159, F = 0.0141).

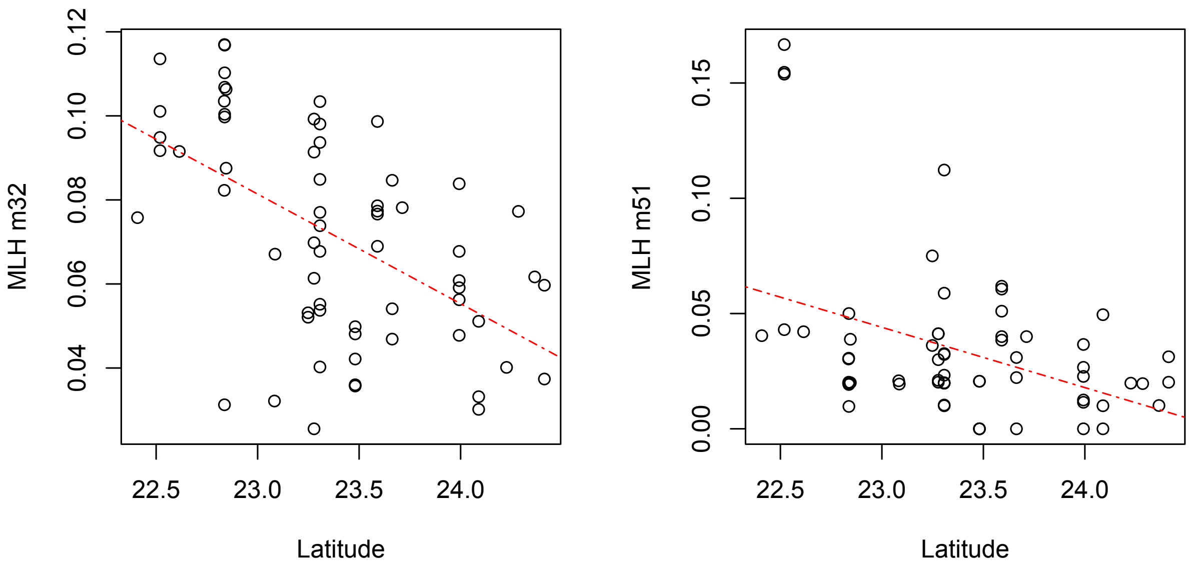

Fig. S4. Scatter plots showing the correlation between the latitudes of the sampling locality and the estimated values of multi-locus heterozygosity (MLH) for each individual sample. Both m32 and m51 datasets revealed a pattern of decreasing in MLH values from southern to northern individuals with statistically significant supports (m32 dataset: $F = 25.28$, $r^2 = 0.2897$, $P = 4.5*10^{-6}$; m51 dataset: $F = 11.91$, $r^2 = 0.1611$, $P = 0.001$).

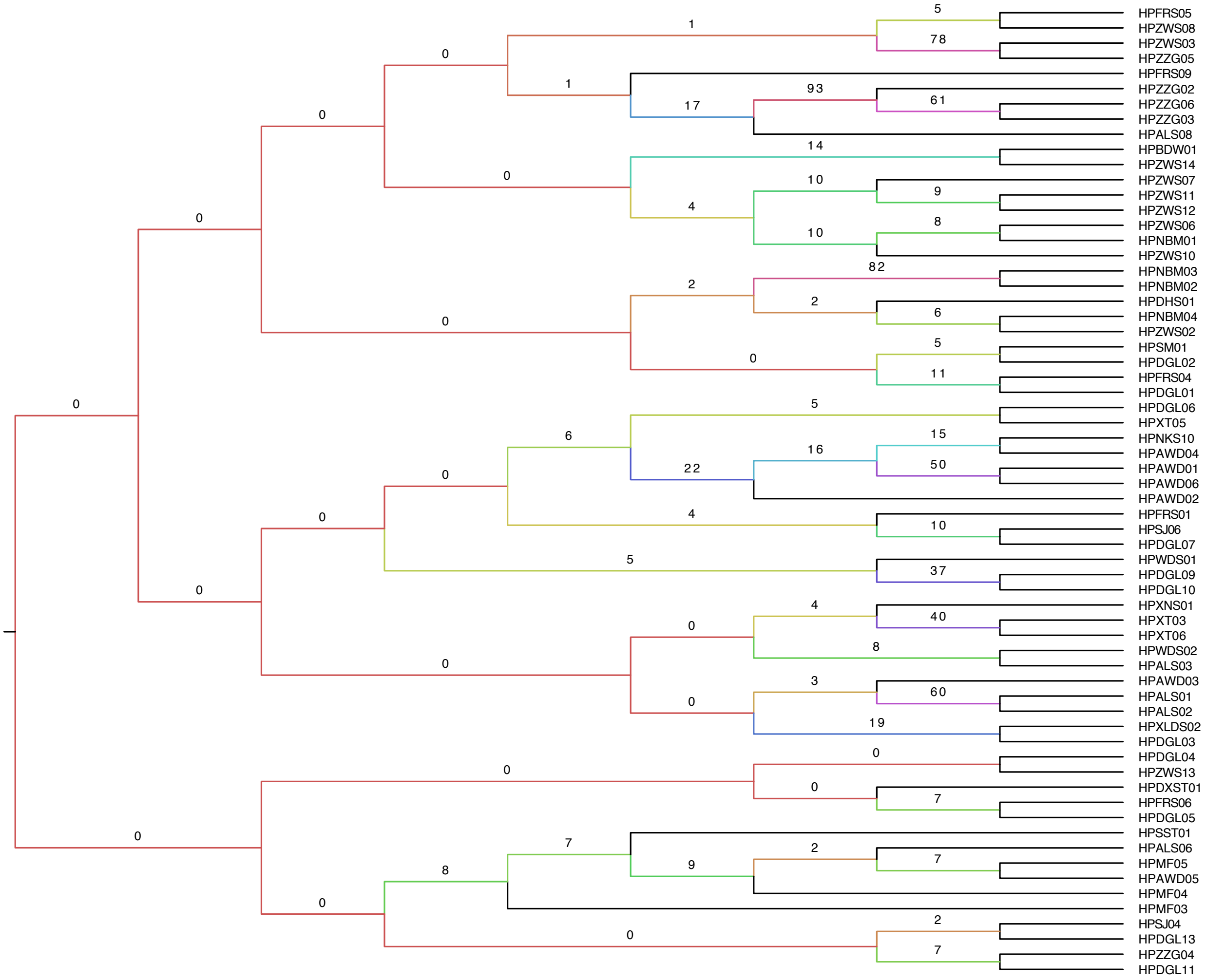

Fig. S5. A midpoint-rooted cladogram based on the maximum likelihood (ML) analysis of the m4 dataset. Branch numbers and colors indicate support values derived from bootstrap analyses.

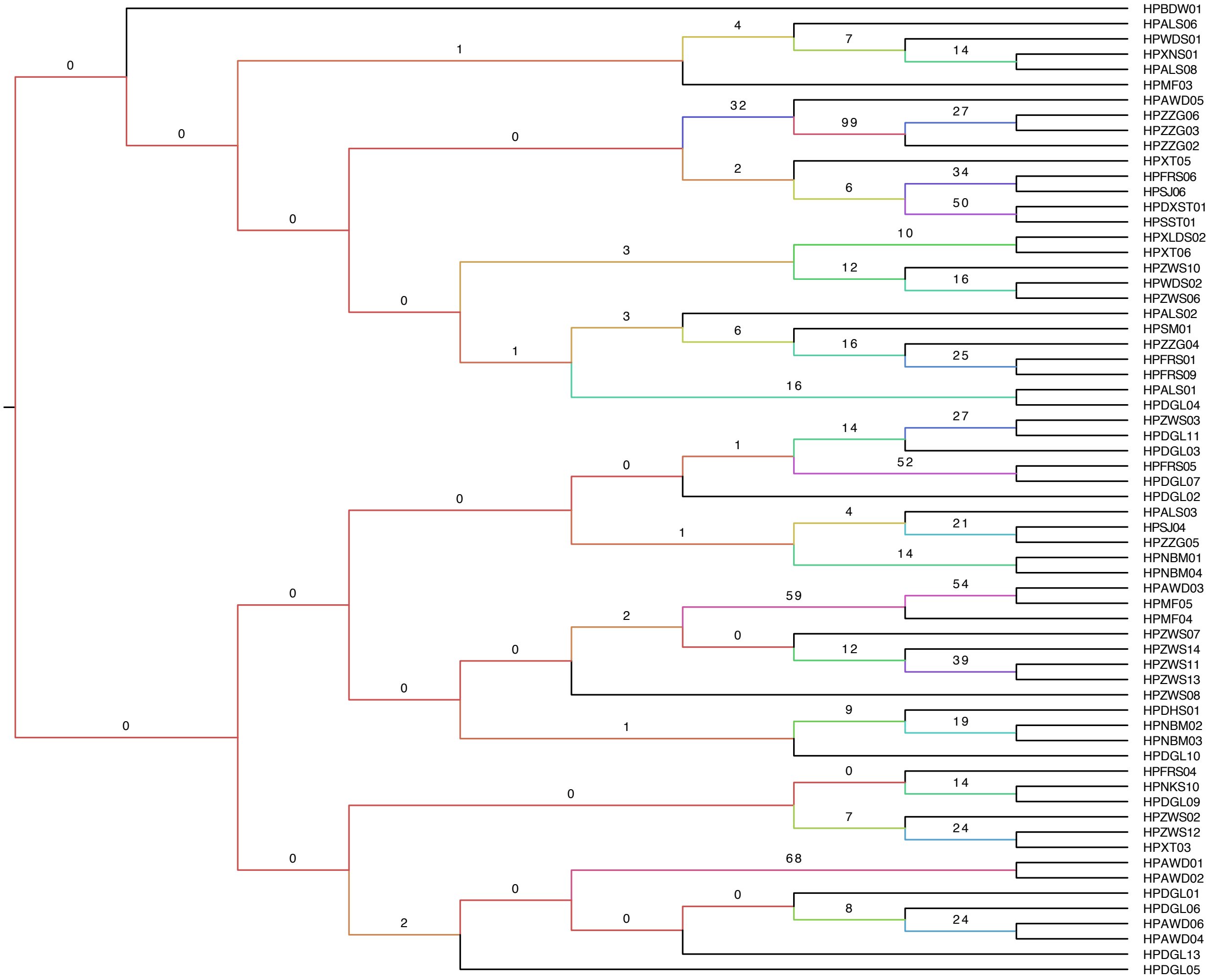

Fig. S6. A midpoint-rooted cladogram based on the maximum likelihood (ML) analysis of the m32 dataset. Branch numbers and colors indicate support values derived from bootstrap analyses.

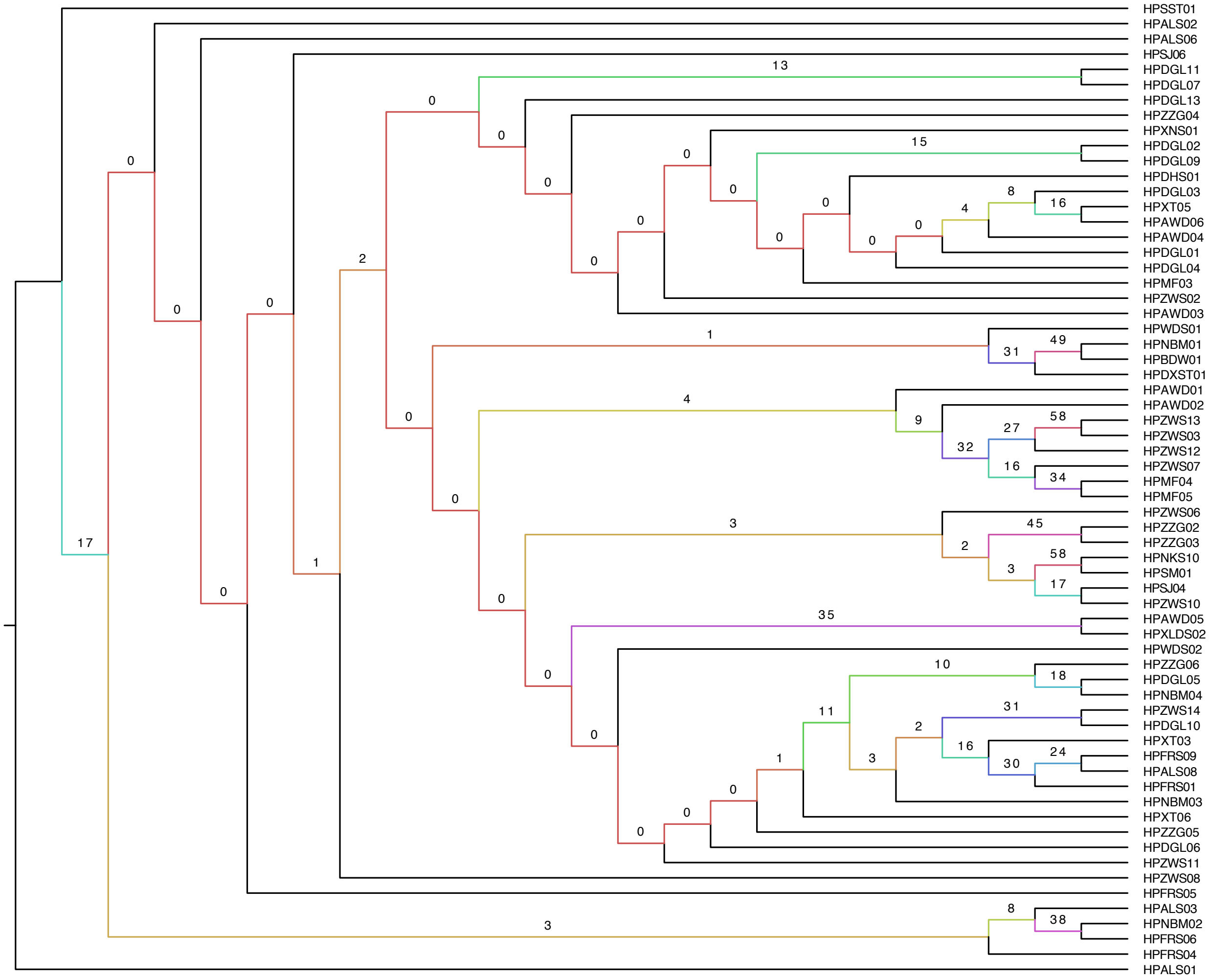

Fig. S7. A midpoint-rooted cladogram based on the maximum likelihood (ML) analysis of the m51 dataset. Branch numbers and colors indicate support values derived from bootstrap analyses.

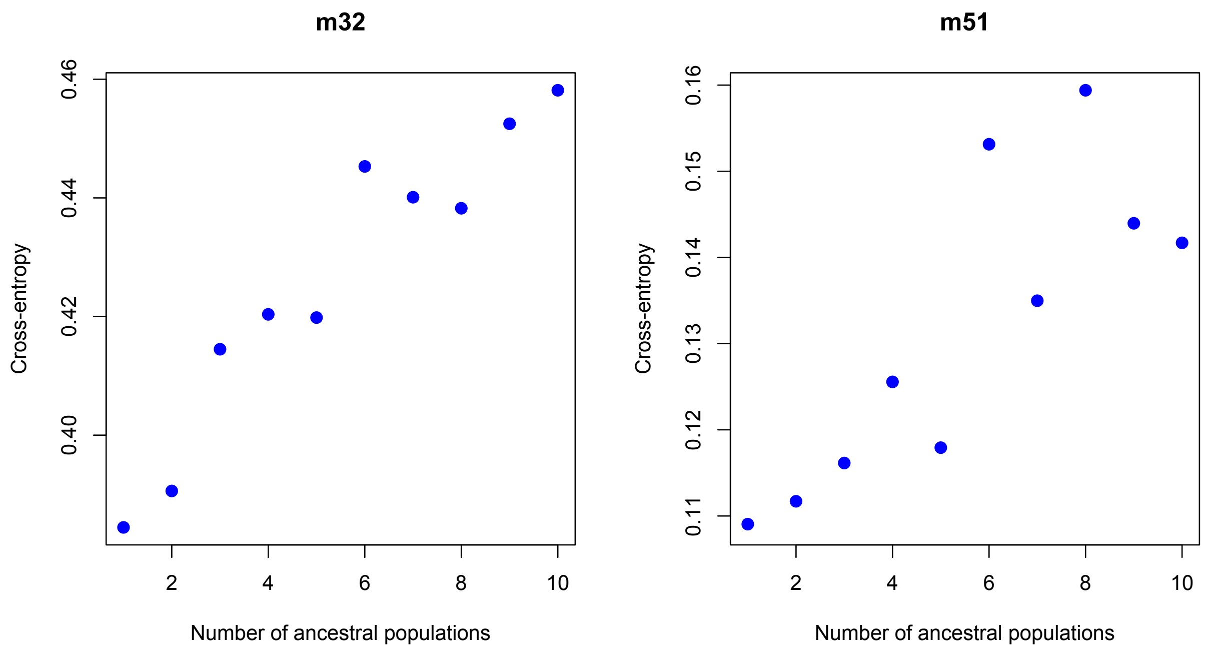

Fig. S8. The estimated number of ancestral populations best explaining the genotypic data was determined using two datasets and an entropy criterion, with 100 cross-validation repetitions. The best number of ancestral population in both datasets are 1.

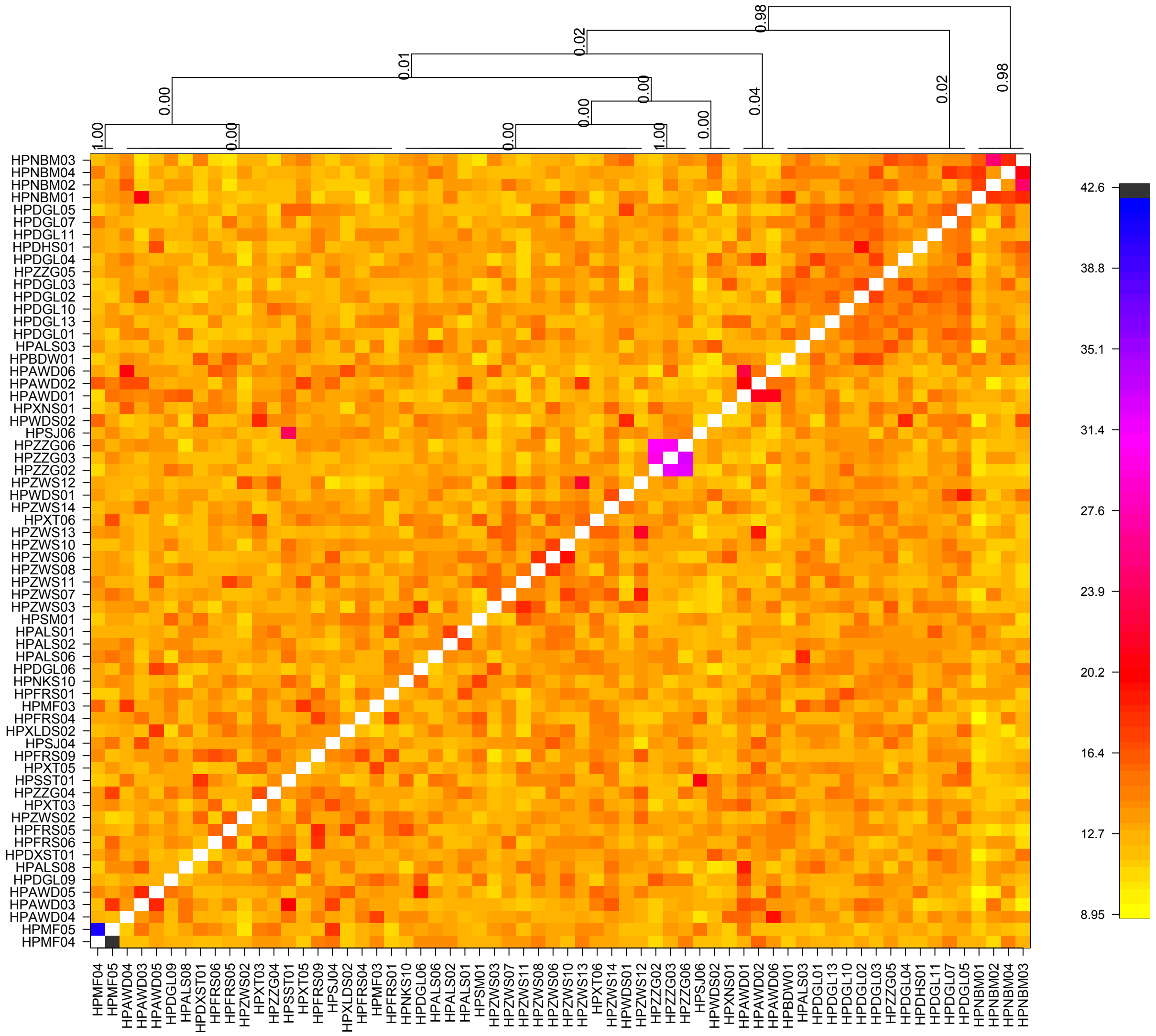

Fig. S9. A co-ancestry plot among the studied samples based on the m32 dataset.

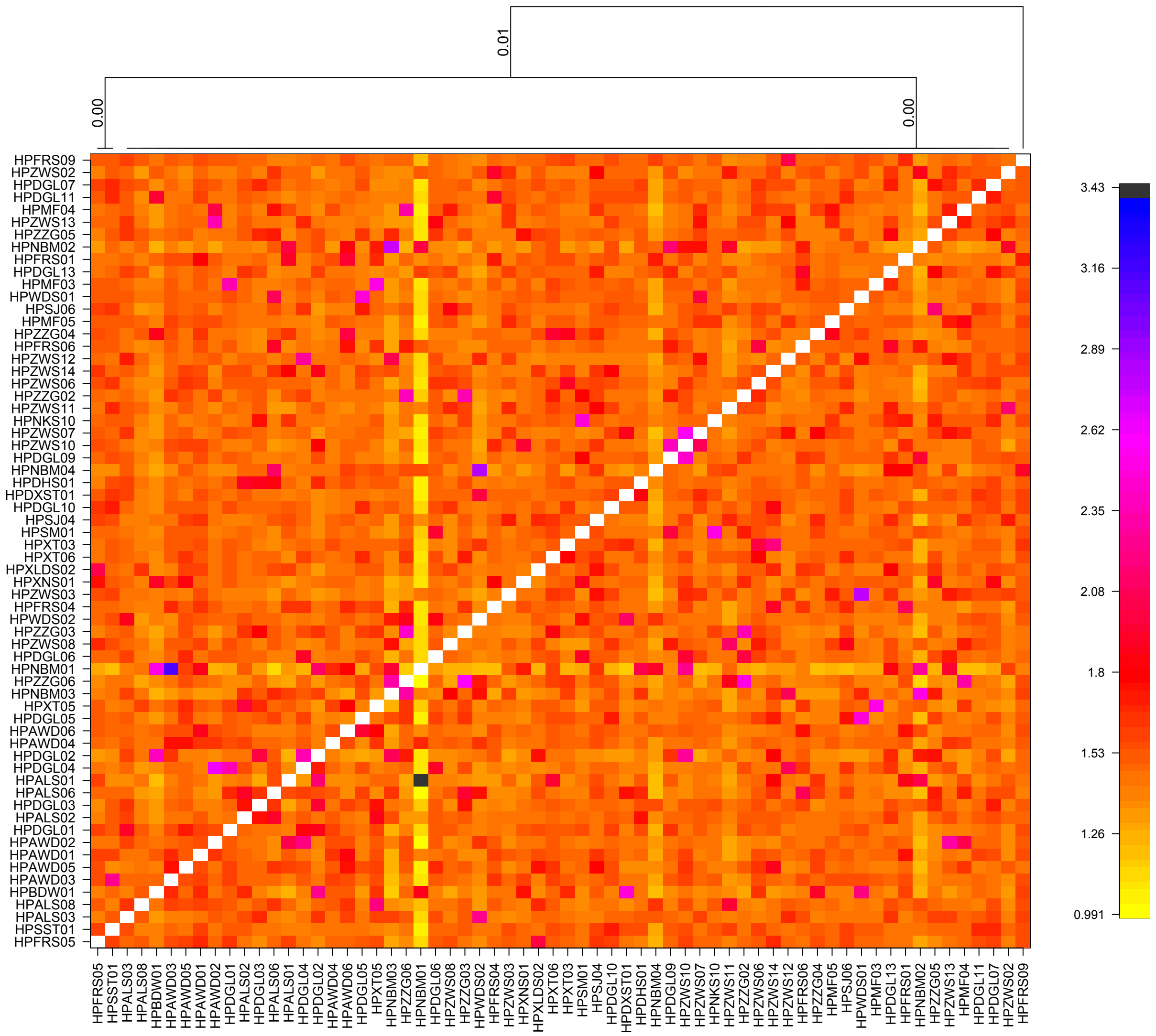

Fig. S10. A co-ancestry plot among the studied samples based on the m51 dataset.

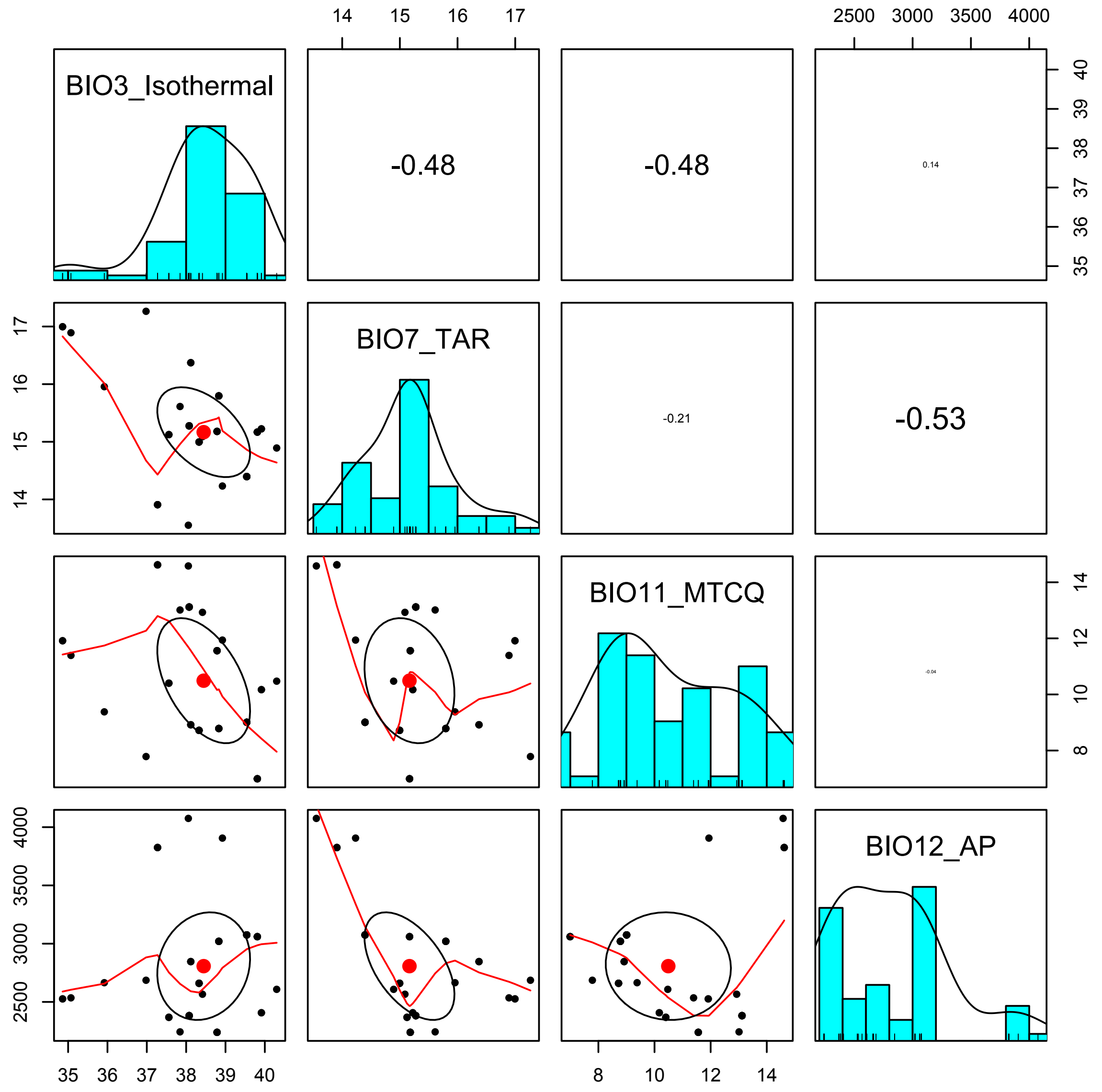

Fig. S11. The four bioclimatic variables used to detect genotype-environment associations in our study. Specifically, they showed no significant correlation among them.

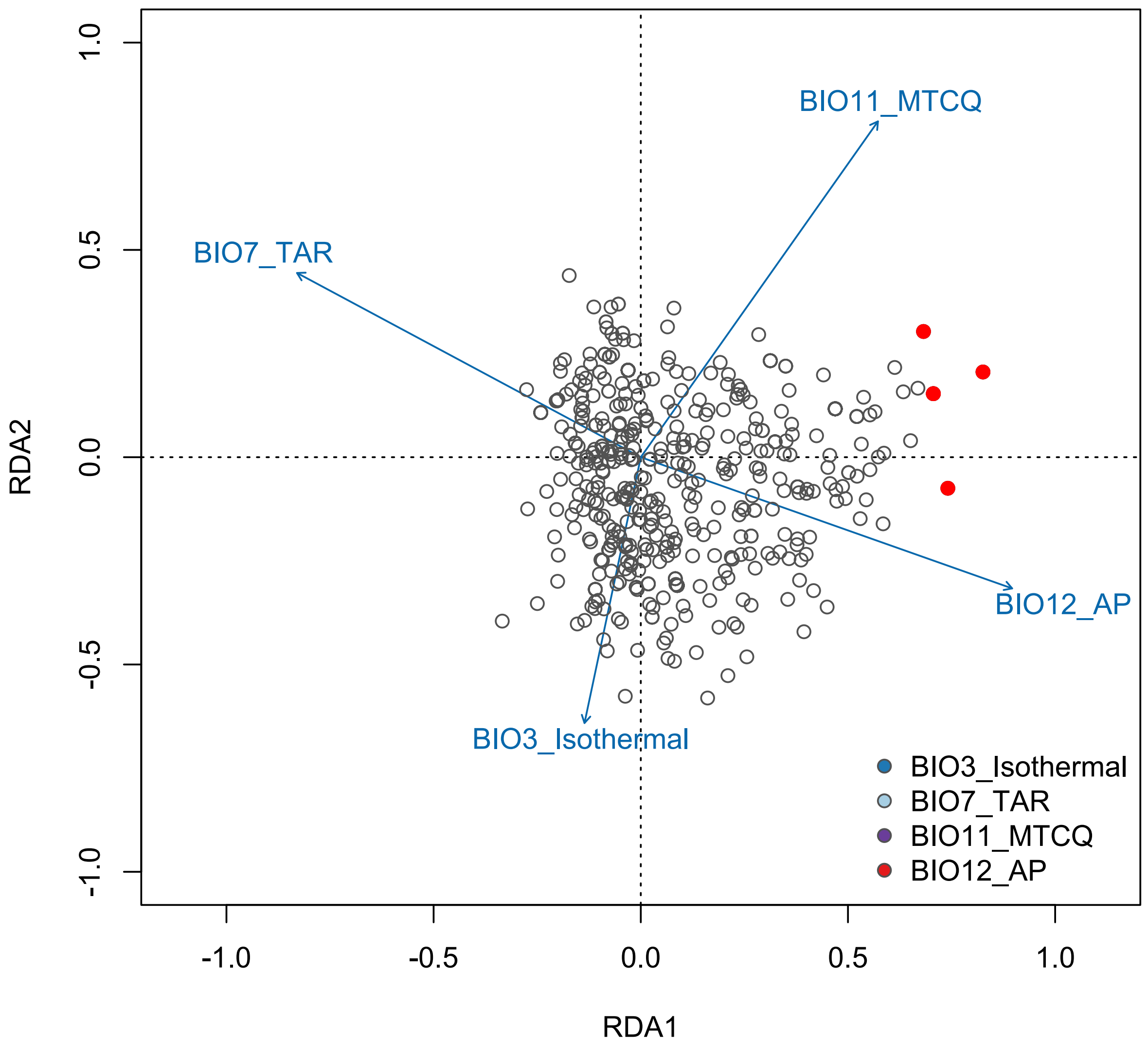

Fig. S12. Outlier loci detected using the m32 dataset from redundancy analysis. Notably, all five loci identified based on a selective criterion of three standard deviations exhibit significant covariation with the annual precipitation bioclimatic variable (BIO12).
